# What is the evidence for dietary modification in the management and prevention of malignant bowel obstruction? A scoping review

**DOI:** 10.1007/s00520-025-09279-y

**Published:** 2025-02-27

**Authors:** Ellie Ware, Laura Tookman, Erin Stella Sullivan, Lina Johansson, Iain McNeish, Lindsey Allan

**Affiliations:** 1 Imperial College Healthcare NHS Trust, London, UK; 2 Department of Surgery & Cancer, Imperial College London, London, UK; 3 Faculty of Medicine, Imperial College London, London, UK; 4 School of Life Course & Population Sciences, Faculty of Life Sciences & Medicine, King’s College London, London, UK; 5 Department of Nutritional Sciences, School of Life Course & Population Sciences, Faculty of Life Sciences & Medicine, King’s College London, London, UK; 6 Royal Surrey NHS Foundation Trust, Guildford, Surrey UK; 7 Department of Nutrition & Dietetics, Imperial College Hospitals NHS Trust, London, W6 8RF UK

**Keywords:** Diet, Fibre, Outcome assessment, Healthcare, Bowel obstruction, Neoplasms

## Abstract

**Purpose:**

Dietary modification is one tool in the multidisciplinary and multi-faceted management of malignant bowel obstruction (MBO). However, the evidence for this has not been systematically explored and no guidelines currently exist. The purpose of this review was to identify the type and breadth of published evidence available to support the use of dietary modification in MBO, and to identify key characteristics of dietary interventions and outcome measures used in evaluating these interventions.

**Methods:**

Systematic searches of three databases were conducted, last in September 2024. Title and abstract screening and full-text review were conducted before data were extracted using a data extraction tool.

**Results:**

Only seven records met the criteria for inclusion. Quality of interventions was low, with four abstracts, one retrospective review and two feasibility studies identified. Most interventions focused on gynaecological cancers, where MBO is most prevalent. Key characteristics of dietary modification included a low-fibre diet and modification of the texture of the diet. These approaches were often used in conjunction and in a stepwise manner (progressing from liquid to soft to low-fibre diet). All records reported benefit of dietary modification, but with limited justification. The number, type and quality of records retrieved might reflect that this is a novel area of research, with local practice and clinical experience being published as abstracts. We found no methodologically robust, large-scale interventions.

**Conclusion:**

This review demonstrates a lack of evidence to support the use of dietary modification in MBO. High-quality studies assessing the efficacy and impact of dietary modification are needed to support the advice commonly being provided in clinical settings. However, this research is ethically and logistically challenging to conduct. Nutritional management guidelines based on expert consensus might be a useful resource for clinicians managing MBO given the lack of research evidence currently available to inform practice.

**Supplementary information:**

The online version contains supplementary material available at 10.1007/s00520-025-09279-y.

## Introduction

Malignant bowel obstruction (MBO) is a common complication in advanced malignancy, particularly in gynaecological and gastrointestinal tumours that spread within the abdominal cavity. MBO affects up to 51% of patients with recurrent gynaecological cancers [1], and malignant obstructions of the small bowel are more prevalent than large bowel obstructions [2].

MBO typically presents as colicky abdominal pain, distention, nausea and bilious vomiting, and an inability to pass wind or stool [3]. This occurs due to either a functional or mechanical obstruction, the aetiology and pathophysiology of which are well described elsewhere [4]. MBO is often recurrent with symptoms increasing in severity and frequency over time, until, without surgical intervention, obstruction becomes a permanent state [3, 5]. This results in frequent, protracted hospital admissions [1, 5], with significant burden both for patients and healthcare services. In addition to diminishing well-being and quality of life (QoL) [3, 5, 6], patients with MBO have reported lack of attention to nutritional problems, fear of starvation and unmet supportive care needs [7–9].

In advanced malignancy and where obstruction is multi-level, surgical input is often not possible and management is therefore conservative. The aim is not to entirely resolve the cause of the obstruction but to allow a period of gut rest giving time to regain some bowel function, and to improve symptom control [10]. Conservative management typically includes insertion of a naso-gastric tube to drain the contents of the stomach, which alleviates pressure, nausea and vomiting. At the same time, intravenous fluids and medications including anti-emetics, pain relief, anti-secretory drugs and steroids are given. Whilst support with clinically assisted nutrition and hydration in the form of parenteral nutrition should be considered, towards the end of life, optimising oral intake where possible is often considered more appropriate [11, 12].

Evidence-based guidelines for the medical management of MBO exist, and reference is made to dietary intervention (see Table 1). Guidelines acknowledge a lack of available evidence to guide the nutritional management of MBO in advanced cancer [4]. Current recommendations focus on the re-introduction of oral intake following an episode of MBO, rather than the prevention of, or minimising the risk of, MBO occurring or re-occurring. There are no existing evidence-based guidelines focusing specifically on nutrition and the role of the diet in the management of MBO. It is common practice for individuals with or at risk of bowel obstruction to follow a low-fibre and/or texture-modified diet to tolerance. Terminology used to describe dietary modification is not standardised and is likely to vary depending on who is providing the advice and when. There is a role for dietary intervention in MBO management, and oral intake is often sub-optimal [13].

**Table 1:** Summary of dietary advice for MBO included within published recommendations and guidelines

Title	Author, publication date	Population	Design	Recommendation	Cited evidence for recommendation
MASCC multidisciplinary evidence‑based recommendations for the management of malignant bowel obstruction in advanced cancer	Madariaga et al 2022 [4]	Malignant bowel obstruction in advanced cancer	Systematic review where outcome measures including symptom control, bowel obstruction resolution, prognosis, survival and quality of life were reported (*n* = 397)	‘When a patient is initially diagnosed with MBO, they should be made Nil Per Os (NPO; nothing by mouth), and then when the acute MBO resolves fully or partially, a symptom led, slow and graded reintroduction to oral diet is recommended. This may include clear fluids, free or full fluids, texture modified low fiber diet (soft, minced, and pureed), and if tolerated, back to normal textured low fiber diet (level of evidence: IV; grade: B)’ ‘Nutrition interventions should be initiated in patients with advanced cancers only where the benefits of these interventions on quality of life and survival outweigh the risks, with clear expectations discussed by a multidisciplinary team with patients and families (level of evidence: IV; grade: B)’	Scottish palliative care guidelines, Health Improvement Scotland, 2024 [14] ESPEN guideline on ethical aspects of artificial nutrition and hydration, 2016 [15] Narrative review, low-residue and low-fiber diets in gastrointestinal disease management, 2015 [16] ESPEN practical guideline: Clinical nutrition in cancer, 2021 [11]
Symptom Control: Bowel Obstruction	Health Improvement Scotland Scottish palliative care guidelines 2024 [14]	Not stated	n/a	‘Nil by mouth Ice to suck Small amounts of food and drink as wanted Low fibre diet’	No evidence cited
Practice guidance on the management of acute and chronic gastrointestinal problems arising as a result of treatment for cancer	Andreyev et al 2011 [17]	Patients with chronic gastrointestinal symptoms related to cancer or cancer treatments	Multidisciplinary literature review	‘Excess fibre in the diet may precipitate subacute obstruction if a stricture is present’ ‘If low-fibre diets are indicated they should be prescribed by a qualified dietitian, should initially be time limited and the clinical benefit from the diet reviewed’ ‘If cancer is present, the nature of the intervention should be influenced by the expected prognosis of the recurrence’	No evidence cited

In summary, the presentation, aetiology and guidelines for medical management of MBO are well established. However, whilst dietary interventions are commonplace within clinical practice, the rationale and evidence for these interventions have not been explored in any existing publication.

### Objectives

The purpose of this scoping review was to identify the type and breadth of published evidence available to support the use of dietary modification in the management of MBO.

1. To identify any published interventions that report and evaluate the use of a dietary intervention in managing MBO
2. To identify key characteristics of any dietary interventions used in published literature
3. To identify the outcome measures used when evaluating the impact or efficacy of a dietary intervention.

## Methods

This review is presented in the format of the Preferred Reporting Items for Systematic Reviews and Meta-analyses Extension for Scoping Reviews (PRISMA-ScR) [18]. The protocol for this scoping review was retrospectively registered (10.17605/OSF.IO/MR8FG).

### Eligibility criteria

Eligibility criteria were structured using the PICOS model [19].

#### Population

Include:

- Participants with or at risk of malignant bowel obstruction.
- Interventions for people with or at risk of malignant bowel obstruction.

Exclude:

- Bowel obstruction not related to malignancy.
- Studies where a diet intervention is mentioned but that are not related to bowel obstruction.
- Animal studies.

#### Intervention

Include:

- Records that include use of dietary modification in the management of bowel obstruction.
- Records that directly relate to or report the use of dietary modification in the prevention or management of bowel obstruction.

Exclude:

- Records where dietary modification is used or referred to but no rationale or further information is included.
- Records where dietary modification is used or referred to but this is not the focus of the study or intervention.

#### Comparator

Include:

- Studies with or without a comparator.

Exclude:

- n/a.

#### Outcome

No other eligibility criteria are listed.

#### Study characteristics

Include:

- Publication in an academic peer-reviewed journal.
- Study population of people with MBO or at risk of MBO.
- Any record that reported an intervention with dietary modification for the management or prevention of MBO.
- Accessible in English language.

Exclude:

- n/a.

### Searches

Systematic searches were conducted using EBSCO (CINAHL and Medline) and Ovid (EMBASE) platforms. Searches were conducted from November 2023 to September 2024, with all results since database inception eligible for inclusion.

A comprehensive search strategy was developed (EW) and is presented in supplementary file 1. Two search concepts, ‘diet’ and ‘obstruction’, were identified. Search terms to describe a dietary intervention, e.g. ‘modified diet’ and ‘texture modified’, or to describe obstruction, e.g. ‘intestinal blockage’, were included. Collated terms for each concept were searched together to identify records that related to both ‘diet’ and ‘obstruction’.

### Procedure

Results were imported to Covidence [20] for review by two authors (EW, LA), and any duplicates were removed. Records were screened for inclusion by title and abstract or by full-text review. If an agreement for inclusion could not be reached, a third reviewer was available to give a majority decision (LJ, ESS).

Reference lists of records reviewed in full were screened for additional relevant records (EW, LA). Included records are listed in supplementary file 2.

### Data extraction

Data were extracted using a template developed by the authors (EW, LA). Due to the design and quality of studies included, for example interventions published as abstracts only, some data were missing and contacted authors did not provide additional detail (see supplementary file 3).

## Results

Searches of three databases retrieved 59 records. Two additional records were included following hand searches and by suggestion of the authors (EW, LA).

**Fig. 1:**
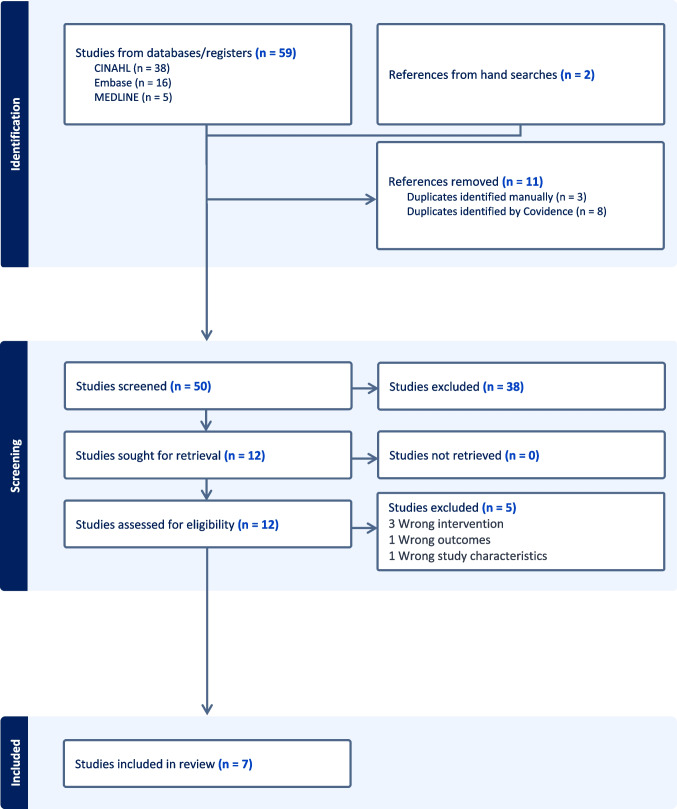
Flow diagram—Preferred Reporting Items for Systematic Reviews and Meta Analysis (PRISMA) [18]

Eleven duplicates were removed. Title and abstracts were screened for 50 records, 38 of which did not meet inclusion criteria. Following full-text review (*n* = 12), seven records were included. Reference lists were screened during the twelve full-text reviews with no further relevant records identified. Records were excluded at full-text review stage due to wrong intervention (*n* = 3), wrong outcomes (*n* = 1) and wrong study characteristics (*n* = 1). Forward searching for citations of included studies returned no further relevant records (see Fig. 1).

Of seven included records, five were published as abstracts [21–25]. One record retrieved in searches was an abstract of a feasibility study [25] subsequently published in full text following initial submission of this review, and was therefore included in full [26]. Other records were a short communication [27], and a feasibility study [28]. All interventions were in high-income, western, English-speaking countries: three from the UK [21, 26, 28], three from the same cancer centre in Canada [22–24] and one from the USA [27].

Three records described interventions for MBO generally, rather than for a specific tumour group [21, 26, 28]. One record referred specifically to cancers of pancreatic origin but concluded that the same dietary advice might be applicable to other conditions affecting the bowel including colorectal and ovarian cancers [24]. The remaining three records from the same institution in Canada stated that their intervention was for gynaecological cancers specifically [22–24].

### Synthesis of results

1. The use and effectiveness of a modified diet in managing MBO.

Three abstracts reported the use of an intervention led by a care team at a large cancer centre in Canada. Bhat et al. [22] described a multi-faceted, proactive intervention that included dietary advice, education and preventing hospital admissions with MBO. Dietary advice was described as ‘full-fluid’ and ‘low fibre diet’, though these terms were not defined. They concluded that care was optimised with their intervention; however, this was not described further. Liu et al. [23] reported preliminary results of the MAMBO trial [29]. This is a prospective, observational study of patients with advanced gynaecological cancer and who are at risk of or who have MBO. Researchers aimed to measure optimisation of multidisciplinary care, measured by time spent in and out of hospital within the first 60 days of a diagnosis of MBO. They described a ‘low fibre’ diet intervention titrated by symptom severity, and unlike Bhat et al. [22], they reported specific outcome measures (see Table 2). However, their results and effectiveness of their intervention were not described within this abstract. They did conclude that patient care and outcomes were improved in the intervention group. In a retrospective analysis of the same intervention, Lee et al. [24] reported reduced hospital length of stay (LoS) and longer median overall survival in the intervention group, who received dietary advice. They also reported a greater number of patients going on to receive further chemotherapy and less patients requiring surgery.

**Table 2:** Summary of dietary interventions, outcome measures and authors conclusions of included records

Title	Author, year, study design or record description	Location	Population	Diet intervention	Outcome measures	Authors conclusions
Four stage low fibre dietary guidance for patients suffering subacute malignant bowel obstruction	Onions and Wilderspin 2021 [21] Conference abstract describing the development of a local dietary intervention for MBO	Worcester, UK	Patients with subacute or resolving malignant bowel obstruction (MBO) (in hospital) (*n* = not stated)	Four staged low-fibre diet plan 1. Clear fluids 2. Liquid low fibre 3. soft low fibre 4. Normal texture low fibre Written information for patients outside of hospital (MBO diet advice booklet)	None reported	Patients reported relief of symptoms Patients are able to stay at home longer and control symptoms better with MBO diet advice booklet
Proactive inter-professional program to manage malignant bowel obstruction (MBO) in women with advanced gynecological cancer: Improving quality of care, education and awareness of malignant bowel obstruction among patients and healthcare providers	Bhat et al 2020 [22] Conference abstract describing an inter-professional management programme for malignant bowel obstruction	Toronto, Canada	Advanced gynaecological cancers (*n* = 404)	Full-fluid or low-fibre diet advice within a symptom-led inter-professional management programme, including a bowel management regime, YouTube videos and written patient education resources Nurse-led intervention	None reported	Care was optimised with implementation of an inter-professional management program Patient-centred approach improved self-management
Risk stratified multidisciplinary ambulatory management of malignant bowel obstruction (MAMBO) program for women with gynecological cancers: Preliminary results from a prospective single-center study	Liu et al 2020 [23] Meeting abstract presenting preliminary results of a single-centre prospective study (MAMBO [29]) Retrospective analysis with historical comparison	Toronto, Canada	Gynaecological cancers (*n* = 70)	Low-fibre diet titrated by severity of symptoms within a multidisciplinary management algorithm for MBO	• Weight • Albumin • Standardised patient-reported outcome measures (PROMs) • Symptoms including fatigue and well-being • Mean number of days in hospital • Chemotherapy received after MBO • Median time from first MBO to death	Patient care and outcomes (LoS and survival) were improved for patients receiving the intervention compared with the historical control group
Optimizing the Care of Malignant Bowel Obstruction in Patients With Advanced Gynecologic Cancer	Lee et al 2019 [24] Retrospective analysis of an inter-professional MBO program	Toronto, Canada	Advanced gynaecologic cancer (*n*=169)	Low-residue diet Low-fibre diet	• Cumulative hospital length of stay (LoS) within 60 days of MBO diagnosis • Interventions received including chemotherapy and surgery	The intervention significantly affects patient care and may improve LoS, survival, avoidance of surgery and palliative chemotherapy rates Patients are empowered to recognise MBO symptoms for early intervention
Management of inoperable malignant bowel obstruction using the 4-step BOUNCED diet*	Allan et al October 2024 [26] Feasibility study	Guildford, UK	Patients with MBO who were able to tolerate oral diet and had 1 or more symptoms of MBO (*n*=26)	4-step bowel obstruction diet: 1. Clear fluids 2. Thin liquids 3. Purée 4. Soft sloppy foods which are low in fibre Education for patients to self-manage oral intake based on bowel obstruction symptoms	• Symptoms of MBO (pain, bloating, early satiety, nausea, vomiting) using • Memorial Sloan Assessment Scale (MSAS) [30, 31] • Quality of life using EORTC-QLQ-C30 [32]	Modified consistency low-fibre diet is easy to follow Symptoms of MBO, admissions to hospital and quality of life (QoL) may be improved with the intervention More research is required
Can a soft diet prevent bowel obstruction in advanced pancreatic cancer?	McCallum et al 2002 [27] Retrospective review published as a short communication	Cleveland, USA	Pancreatic cancers with no prior history of intestinal obstruction (*n*=17)	Gastrointestinal (GI)/soft diet - Chew food thoroughly - Do not eat raw fruit or vegetables – only canned or cooked - Do not eat green peas, cooked dry beans, peas or lentils - Do not eat nuts, popcorn, seeds, or whole spices Delivered by registered dietitian	• Incidence of intestinal obstruction prior to death	Gastrointestinal (GI)/soft diet prevents obstruction in pancreatic cancer patients This intervention might prevent bowel obstruction in other groups, e.g. colorectal, abdominal, ovarian cancers and after pelvic radiation Intervention might reduce morbidity, mortality, reduce healthcare costs and be beneficial for QoL
Can we improve the management of inoperable malignant bowel obstruction? Results of a feasibility study of elemental diet as an alternative to parenteral nutrition in patients with advanced gynaecological cancer	Allan et al August 2024 [28] Feasibility study	Guildford, UK	Advanced gynaecological cancer (*n*=19)	Elemental diet – Elemental 028 Extra Liquid (Nutricia) 250 ml carton containing 215 kcal, 6.3 g protein Number of cartons recommended was individualised and not reported *Elemental diet is described as a liquid diet containing protein as amino acids and fat as medium chain triglycerides. It is absorbed in the upper-small intestine and indications for use include inflammatory bowel disease, short bowel syndrome and malabsorption [28]	• Tolerance and acceptability of elemental diet • Quality of life using EORTC-QLQ-C30 [32] • MBO symptoms (vomiting, pain, bloating) using Memorial Sloan Assessment Scale (MSAS) [30, 31] • Weight change • Receiving further chemotherapy • Nutritional intake • Patient perspective	Symptoms of MBO were not exacerbated with elemental diet Elemental diet may improve quality of life More research is required

*Full-text publication of conference abstract identified in systematic searches

McCallum et al. [27] published a retrospective review whereby 17 patients received dietary advice from a dietitian. They described a ‘gastrointestinal (GI)/soft diet’ (see Table 2) and compared outcomes with a control group who received no specific dietary advice (*n*=17). Prior to death, no patients in the dietary intervention group had experienced MBO, whilst 12 of 17 patients who received no dietary intervention did so (*p*=0.001). They concluded that a ‘gastrointestinal (GI)/soft diet’ prevented MBO in pancreatic cancers and deduced that this may also be beneficial in other malignancies. However, it is not possible to determine causality given the study was retrospective and participants were not randomised. The author’s conclusion that the intervention can be transferred to other tumour groups remains questionable without further trials.

In an abstract, Allan et al. presented preliminary results of a feasibility study [25]. This study was subsequently published in full, with authors aiming to investigate the use and effectiveness of a 4-step bowel obstruction diet [26]. With a prospective, mixed-methods, non-randomised study design, the authors measured the acceptability and impact on MBO symptoms with their diet intervention. Patients with ovarian and colorectal cancers were included. Results included a reduction in pain (from 96 to 63% on days 1 and 28 respectively (*p*=0.004)), improved patient-reported quality of life (*p*≤0.001) and significant reduction in number of admissions (*p*=0.018) and bed days (*p*=0.004).

Onions and Wilderspin [21] described implementation of a ‘4-step bowel obstruction diet’, which was based, with permission, on the dietary intervention described above [25, 26]. They did not report any objective outcome measures, but concluded patient-reported improvement in symptoms resulting from their intervention, without further elaboration.

In a feasibility study, 29 women with a gynaecological malignancy and MBO, able to tolerate oral liquids, were recruited (aged 35–91, median age 75.5 years). Elemental liquid diet (Elemental 028 Extra (E028), Nutricia Ltd) was given as an alternative to parenteral nutrition. E028 is a nutritional supplement drink licenced for use in gastrointestinal diseases such as Crohn’s disease. Rationale for giving an elemental liquid diet within this cohort was that it is absorbed in the upper intestine. However, its use is not routine practice in MBO. The authors reported a reduction in symptoms of MBO after 15 days, including pain and vomiting, and an improvement in QoL. They concluded that elemental diet may be effective in those who can tolerate oral liquid diet [28].

2. Characteristics of published dietary interventions used in MBO.

In the seven records identified, two elements of diet modification were commonly reported, reducing fibre and texture-modified diet. Six records reported using both a low-fibre and texture-modified diet [21–24, 26, 27]. McCallum et al. [27] referred to a ‘gastrointestinal (GI)/soft diet’, and whilst their intervention did not state ‘low fibre’, restricted foods were higher in fibre (see Table 2).

Dietary modification to reduce fibre is described as a ‘low-fibre’, ‘low-residue’ or a ‘gastrointestinal (GI)’ diet, demonstrating a variation in terminology used. Advice to modify the texture of the diet included ‘liquid’ and ‘soft diet’, as well as a 4-step approach from clear fluids to solid food that is still soft and low in fibre. One intervention stated specific foods to avoid [27]. A ‘4-stage’ or ‘4-step’ approach is reported across interventions [21, 26].

In a feasibility study, Allan et al. [28] used E028, the only study of its kind reporting the use of elemental diet as a tool in the management of MBO. The authors did not report other oral dietary intake or nutrition support during the study period, or whether participants received any additional dietary advice, including texture or fibre modification.

3. Outcome measures.

Outcome measures were often stated, but results not published within an abstract. Several records allude to improved patient outcomes or care, but without further elaboration [23, 24].

### Time in hospital

Three records reported time in hospital as an outcome measure [23, 24, 26]. Lee et al. [24] reported a significantly shorter cumulative LoS within the first 60 days of MBO in their intervention group (average 13 days, range 10–16 days, 95% CI) compared to their control group (average 22 days, range 18–26 days, 95% CI, *p*=0.006). Allan et al. [26] reported a reduction in bed days during the intervention period compared to 28 days prior (45 to 2 days respectively, *p*=0.004). Liu et al. [23] reported a reduction in mean number of days in hospital prior to and during the intervention period (18 days and 10 days respectively, *p*=0.009).

### Symptoms

Change in symptoms of MBO were measured using validated questionnaires (Memorial Sloan Assessment Scale (MSAS) [30, 31] and reported by Allan et al. [26, 28]. Patient-reported symptoms improved with the 4-step low-fibre and texture-modified diet and elemental diet. Liu et al. [23] reported fatigue and well-being having greatest impact on QoL; however, results are yet to be published. Onions and Wilderspin [21] concluded that patient-reported symptoms improved, but did not specify which symptoms were measured. Lee et al. [24] concluded that patients in their intervention group were empowered to recognise MBO symptoms earlier, but did not report symptoms as an outcome measure.

### Quality of life

Allan et al. [26, 28] were the only authors who measured QoL with a validated questionnaire (EORTC-QLQ-C30 [32]) as an outcome measure. Both studies reported improved QoL within their intervention groups, but did not elaborate.

### Mortality

Lee et al. [24] reported time from first episode of obstruction to death, demonstrating median increased survival in the intervention group compared with a historical control group (108 to 219 days respectively, *p* = 0.007). Only McCallum et al. [27] reported incidence of intestinal obstruction prior to death with and without dietary intervention, and demonstrated a significant reduction in obstruction within their intervention group (*p* = 0.001).

### Economic outcomes

Two studies commented on reduced LoS or cost saving associated with dietary intervention. The intervention reported by McCallum et al. [27] estimated the average cost and LoS of an admission with MBO was $7500 and 13 days respectively, whereas cost of dietary instruction was $185, suggesting that dietary intervention, if associated with reduced LoS, may be beneficial for both economic and QoL outcomes. Liu et al. [23] demonstrated a reduced LoS for MBO admissions in their intervention group compared with historical controls (*p* = 0.009).

Bhat et al. [22] did not specifically state economic impact; however, they concluded that their intervention optimised the care of patients with advanced gynaecological cancers. In their conclusions, Onions and Wilderspin [21] alluded to the fact that patient information resources can help patients to be in control of their symptoms and prevent hospital admissions, though no outcome evaluation was included.

Managing oral diet following a diagnosis of subacute malignant bowel obstruction: Results of the 4-step BOUNCED diet feasibility study [25]

## Discussion

### Use and effectiveness

This review identifies a lack of high-quality research or evidence for the recommendation of dietary modification in the management of MBO. Whilst consistent results and conclusions in the few published items that were identified suggest that there is benefit in a texture-modified and/or reduced fibre diet in MBO, it is difficult to evaluate the quality of evidence within abstracts, without full-text articles and where confounding variables have not been controlled for. This does not however mean that dietary modification is not beneficial as a tool in the multidisciplinary and holistic management of MBO, rather that further research is needed.

In practice, designing a trial whereby individuals either at risk of or with previous episodes of MBO are randomised to receive different dietary interventions (or no dietary intervention) would be ethically and logistically challenging. The impact of variables such as disease state, anti-cancer treatments, and symptom control measures including medications would need to be considered. In the current literature, these confounding variables have not been controlled for, which is a significant weakness of using patient-reported symptoms, QoL and time in hospital as sole outcome measures. Future studies should incorporate both patient-reported outcomes and clinical characteristics, which can be considered in statistical models. Similarly, qualitative research exploring the lived experience of people with MBO would be valuable, and would help shape the design of patient-centred dietary interventions.

Within the publications identified, the focus of dietary interventions is often the re-introduction of oral intake following an episode of subacute MBO. No studies have explored the actual basis for recommending a low-fibre or texture-modified diet. There is no research to date investigating the role of early diet modification in preventing or delaying MBO, or when or how dietary advice should be provided.

Within the scope of this review, unpublished and non-academic literature and resources were not included. A systematic review of such resources and the dietary advice that they recommend would shed light on current clinical practice within the UK and internationally.

No guidelines for the dietary management of MBO currently exist. In view of the lack of evidence from published studies, an expert-consensus, best-practice guideline would be of benefit to those caring for and providing advice to those with MBO. This could bring together the expertise and clinical experience of dietitians and other members of the care team to standardise clinical practice and resources available to patients and the public. Patient and public involvement in the development of new guidance would ensure this meets the needs of the service user. Future interventions could then compare newly developed interventions to standard care. Currently, there is no defined standard of care, making inter-study comparison challenging.

Dietary modification commonly consists of two approaches, texture modification and reducing dietary fibre, which are often used in conjunction. However, the role and efficacy of a low-fibre compared with texture-modified diet have not been explored. Research identifying whether one approach is more effective than the other, or whether a combination is required, would be novel and could potentially avoid unnecessary restrictions being recommended. This is particularly important with advanced malignancy where prognosis is often short and QoL should be a priority [4]. Restrictive diets may reduce nutritional intake and subsequently increase the risk of malnutrition in an already vulnerable population group. Further research in MBO assessing the impact of these interventions on nutritional status and QoL would be valuable.

Some of the records identified suggest that dietary intervention in MBO can result in reduced LoS [21, 24, 26] or financial savings [23, 24] and more work is needed to establish the potential cost benefits to healthcare providers.

Existing guidelines for the medical management of MBO suggest a multidisciplinary, holistic approach. Despite limited evidence, dietary modification is a routine component of the multi-faceted management of MBO, particularly in advanced malignancy where symptom control and maintaining bowel function to allow individuals to eat and drink become a priority. Where dietary modification advice is required, individuals should be referred to a registered dietitian for support [4, 17, 35].

### Quality of items retrieved

The majority of items identified were abstracts and therefore did not present full results or outcome measures. Existing interventions are small, local interventions, which support the notion that dietary modification is assumed standard practice but without strong evidence. This further demonstrated the need for robustly designed clinical trials rather than a reliance on research-in-practice.

### Terminology

Lee et al. [33] commented that there are no widely agreed definitions of a low-fibre or low-residue diet, contributing to variation in clinical practice. The terminology used to describe dietary interventions for MBO varies widely, which was anticipated and accounted for in the search strategy described in the methodology and presented in supplementary file 1. None of the items identified in this review defined what constitutes a low-fibre diet. MASCC guidelines refer to a low-fibre diet as containing < 10 g fibre/day [4], and reason that lowering dietary fibre results in reduced stool bulk thereby minimising the symptoms of subacute bowel obstruction.

Dietary intervention often refers to texture modification. This advice is also used in the management of dysphagia, using the International Dysphagia Diet Standardisation Initiative (IDDSI) [34]. None of the literature we found refers to this framework, and it is worth noting that similar terminology used for MBO and dysphagia could cause confusion, with implications for patient safety. Standardising both advice provided as well as terminology used to describe this advice may improve care.

### Non-malignant bowel obstruction

Several studies support the use of a low-fibre diet in non-malignant disease where intestinal structures are present, for example in inflammatory bowel disease [17, 33, 36]. Guidelines from the British Society of Gastroenterology recommend that patients with stricturing Crohn’s may need to alter their intake of fibre [37]. Similarly, guidelines from the British Dietetic Association state that dietary fibre is contraindicated in stricturing Crohn’s disease, despite there being no strong evidence from clinical trials for this recommendation [33, 38, 39].

It may seem logical reasoning to apply the same dietary modification recommendations to MBO where presentation and symptoms of obstruction may be similar, or where disease may result in a similar narrowing within the bowel lumen. However, the goals of care are significantly different in those with MBO secondary to advanced disease compared with goals of care in non-malignant bowel obstruction. Efficacy and safety of the transfer of advice from one condition to another are not established. Additionally, considering dietary fibre as a singular substance rather than a heterogeneous group of substances does not adequately consider properties such as solubility, which is likely to affect tolerance and risk of obstruction in MBO.

### Strengths and limitations

Whilst effort was made to capture all language that might be used to define the concepts of diet and obstruction, it is possible that searches did not capture all relevant results. Due to the anticipated lack of published literature, a scoping review was conducted rather than a systematic review, using the PRISMA-ScR format [18]. Data charting and extraction were limited and inter-study comparisons challenging. Several items did not present full details of interventions reported within the scope of an abstract, or report full results or outcome measures.

There is a wealth of non-academic literature available publicly online regarding diet, cancer and bowel obstruction. Information produced by international healthcare providers, charities and patient advocacy groups, businesses, social media content and personal blogs aimed at those with MBO has not been included within the scope of this review, given that the abundance of misinformation and non-evidenced dietary advice relating to nutrition and cancer is well recognised [40, 41]. Whilst a scoping review of this information would be valuable and has not been published to date, the aim of the present review was to evaluate evidence rather than current practice, and therefore only literature published in scientific journals was within the scope of this review. Similarly, the role, appropriateness and ethics surrounding clinically assisted nutrition and hydration in advanced malignancy are much debated within existing literature, but are beyond the scope of this review.

## Conclusions

This scoping review has highlighted a significant lack of relevant, high-quality studies providing evidence for the recommendation of dietary modification in the management of MBO. This reflects the challenges of designing an ethically sound clinical trial and controlling for confounding variables such as disease state, anti-cancer treatments and palliative care interventions. Despite this, dietary modification is a widely used approach, is referred to in clinical management guidelines and is common particularly in re-introduction of oral intake after a subacute bowel obstruction. Whilst we have found no strong evidence to support the use of a modified diet, and developing ethical, adequately powered, well-designed clinical trials in this field remains challenging, this does not mean that dietary modification is not beneficial in the multidisciplinary and holistic management of MBO.

## Supplementary information

Below is the link to the electronic supplementary material.

Supplementary file1 (DOCX 34 KB)

Supplementary file2 (RTF 45 KB)

Supplementary file3 (DOCX 24 KB)

## Data Availability

No datasets were generated or analysed during the current study.
