# Supplementary material for: What is the evidence for dietary modification in the management and prevention of malignant bowel obstruction? A scoping review
Source: Support Care Cancer. 2025 Feb 27;33(3):231. doi: 10.1007/s00520-025-09279-y (PMC11868329; doi:10.1007/s00520-025-09279-y)
Supplement: Supplementary file 1 — Supplementary file1 (DOCX 34 KB) [file 520_2025_9279_MOESM1_ESM.docx]

**What is the evidence for dietary modification in the management and prevention of malignant bowel obstruction? A scoping review**

Supplementary file 1 – Search terms

*Table 1 – Search terms*

| **EBSCO (CINAHL, Medline)** | | |
| --- | --- | --- |
|  | **Phrase ('diet')** | **Search code** |
|  | modified diet | modif* N3 diet* |
|  | dietary modification | diet* N3 modif* |
|  | texture modified | texture N3 modif* |
|  | low fibre | "low* fibre" |
|  | low fiber | "low* fiber" |
|  | reduced fibre | reduce* N3 fibre |
|  | reduced fiber | reduce* N3 fiber |
|  | dietary fibre | diet* N3 fibre |
|  | dietary fiber | diet* N3 fiber |
|  | soft diet | soft* N3 diet* |
|  | liquid diet | liquid* N3 diet* |
|  | fluid diet | fluid* N3 diet* |
|  | sloppy diet | "sloppy diet*" |
|  | soft food | soft* N3 food* |
|  | altered texture | alter* N3 texture* |
|  | altered consistency | alter* N3 consistenc* |
|  | modified consistency | modif* N3 consistenc* |
|  | **Phrase ('obstruction')** | **Search code** |
|  | bowel obstruction | bowel* adj4 obstruct* |
|  | intestinal obstruction | intestin* adj4 obstruct* |
|  | intestinal blockage | intestin* adj4 block* |
|  | intestinal failure | intestin* adj4 fail* |
|  | bowel blockage | bowel* adj4 block* |
| **OVID (EMBASE)** | | |
|  | **Phrase ('diet')** | **Search code** |
|  | modified diet | modif* adj4 diet* |
|  | dietary modification | diet* adj4 modif* |
|  | texture modified | texture adj4 modif* |
|  | low fibre | "low* fibre" |
|  | low fiber | "low* fiber" |
|  | reduced fibre | reduce* adj4 fibre |
|  | reduced fiber | reduce* adj4 fiber |
|  | dietary fibre | diet* adj4 fibre |
|  | dietary fiber | diet* adj4 fiber |
|  | soft diet | soft* adj4 diet* |
|  | liquid diet | liquid* adj4 diet* |
|  | fluid diet | fluid* adj4 diet* |
|  | sloppy diet | "sloppy diet*" |
|  | soft food | soft* adj4 food* |
|  | altered texture | alter* adj4 texture* |
|  | altered consistency | alter* adj4 consistenc* |
|  | modified consistency | modif* adj4 consistenc* |
|  | **Phrase ('obstruction')** | **Search code** |
|  | bowel obstruction | bowel* adj4 obstruct* |
|  | intestinal obstruction | intestin* adj4 obstruct* |
|  | intestinal blockage | intestin* adj4 block* |
|  | intestinal failure | intestin* adj4 fail* |
|  | bowel blockage | bowel* adj4 block* |

*Table 2 – Example search string*

| **Search platform** | **Keywords** | **Number of search results** | **Number of reports included** |
| --- | --- | --- | --- |
| EMBASE | modif* adj4 diet* OR diet* adj4 modif* OR texture adj4 modif* OR "low* fibre" OR "low* fiber" OR reduce* adj4 fibre OR reduce* adj4 fiber OR diet* adj4 fibre OR diet* adj4 fiber OR soft* adj4 diet* OR liquid* adj4 diet* OR fluid* adj4 diet* OR "sloppy diet*" OR soft* adj4 food* OR alter* adj4 texture* OR alter* adj4 consistenc* OR modif* adj4 consistenc*  AND  bowel* adj4 obstruct* OR intestin* adj4 obstruct* OR intestin* adj4 block* OR intestin* adj4 fail* OR bowel* adj4 block* |  |  |
