# Supplementary material for: What is the evidence for dietary modification in the management and prevention of malignant bowel obstruction? A scoping review
Source: Support Care Cancer. 2025 Feb 27;33(3):231. doi: 10.1007/s00520-025-09279-y (PMC11868329; doi:10.1007/s00520-025-09279-y)
Supplement: Supplementary file 2 — Supplementary file2 (RTF 45 KB) [file 520_2025_9279_MOESM2_ESM.rtf]

What is the evidence for dietary modification in the management and prevention of malignant bowel obstruction? A scoping review 
Supplementary file 2 – included items

Allan, L., Hatchett, N., Skene, S.S., Eastley, K.B., Michael, A., 2025. Management of inoperable malignant bowel obstruction using the 4‐step BOUNCED diet. J Human Nutrition Diet 38, e13388. https://doi.org/10.1111/jhn.13388
Allan, L.L., Skene, S.S., Eastley, K.B., Herbertson, R., Smith, E., Michael, A., 2024. Can we improve the management of inoperable malignant bowel obstruction? Results of a feasibility study of elemental diet as an alternative to parenteral nutrition in patients with advanced gynaecological cancer. Supportive care in cancer : official journal of the Multinational Association of Supportive Care in Cancer 32, 567. https://doi.org/10.1007/s00520-024-08709-7
Allan, L., Michael, A., Skene, S.S., Hatchett, N., 2023. Managing oral diet following a diagnosis of sub-acute malignant bowel obstruction: Results of the 4-step bounced diet feasibility study. Clinical Nutrition ESPEN 58, 442. https://doi.org/10.1016/j.clnesp.2023.09.053
Bhat, G., Jivraj, N., Oza, A.M., Dhani, N., Lee, Y.C., Madariaga, A., Kasherman, L., McMullen, M., Liu, S., Bowering, V., Ferguson, S.E., Croke, J., Lheureux, S., 2020. Proactive inter-professional program to manage malignant bowel obstruction (MBO) in women with advanced gynecological cancer: Improving quality of care, education and awareness of malignant bowel obstruction among patients and health care providers. Gynecologic Oncology 159, 55–56. https://doi.org/10.1016/j.ygyno.2020.06.116
Lee, Y.C., Jivraj, N., Wang, L., Chawla, T., Lau, J., Croke, J., Allard, J.P., Stuart-McEwan, T., Nathwani, K., Bowering, V., Karakasis, K., O'Brien, C., Shlomovitz, E., Ferguson, S.E., Buchanan, S., Ng, P., Cyriac, S., Tinker, L., Dhani, N., Oza, A.M., Lheureux, S., 2019. Optimizing the Care of Malignant Bowel Obstruction in Patients With Advanced Gynecologic Cancer. JOP 15, e1066–e1075. https://doi.org/10.1200/JOP.18.00793
Liu, S.L., Lee, Y.C., Jivraj, N., Bowering, V., Wang, L., Bhat, G., Madariaga, A., Kasherman, L., Nathwani, K., Tesfu, A., Lee, S., Ferguson, S.E., Croke, J.M., O'Brien, C., Lau, J., Chawla, T., Schlomovitz, E., Oza, A.M., Lheureux, S., 2020. Risk stratified multidisciplinary ambulatory management of malignant bowel obstruction (MAMBO) program for women with gynecological cancers: Preliminary results from a prospective single-center study. JCO 38, 6062–6062. https://doi.org/10.1200/JCO.2020.38.15_suppl.6062
McCallum, P., Walsh, D., Nelson, K.A., 2002. Can a soft diet prevent bowel obstruction in advanced pancreatic cancer? Support Care Cancer 10, 174–175. https://doi.org/10.1007/s005200100307
Onions, S., Wilderspin, N., 2021. P-136 Four stage low fibre dietary guidance for patients suffering subacute malignant bowel obstruction, in: Poster Presentations. Presented at the A New World – Changing the landscape in end of life care, Hospice UK National Conference, 3–5 November 2021, Liverpool, British Medical Journal Publishing Group, p. A58.2-A58. https://doi.org/10.1136/spcare-2021-Hospice.153
