# Supplementary material for: What is the evidence for dietary modification in the management and prevention of malignant bowel obstruction? A scoping review
Source: Support Care Cancer. 2025 Feb 27;33(3):231. doi: 10.1007/s00520-025-09279-y (PMC11868329; doi:10.1007/s00520-025-09279-y)
Supplement: Supplementary file 3 — Supplementary file3 (DOCX 24 KB) [file 520_2025_9279_MOESM3_ESM.docx]

**What is the evidence for dietary modification in the management and prevention of malignant bowel obstruction? A scoping review**

Supplementary file 3 - Data extraction table

| **General Information** | |
| --- | --- |
| Study ID |  |
| Title |  |
| **Authors and Publication** | |
| Authors |  |
| Lead author contact details |  |
| Year of publication |  |
| Location of publication |  |
| **Study characteristics** | |
| Methods |  |
| Aim of study |  |
| Study design |  |
| Start date |  |
| End date |  |
| **Population description** | |
| Participants |  |
| Total number of participants |  |
| Inclusion criteria |  |
| Exclusion criteria |  |
| Intervention |  |
| Description |  |
| Outcome measures |  |
| **Results** | |
| Results |  |
| **Recommendations** | |
| Recommendations |  |
| **Conclusion** | |
| Conclusion |  |
